# Lifetime Prevalence of Betel Nut Chewing in India and Taiwan: Raising Awareness of Oral Cancer Risks and the Urgent Call for Regulation

**DOI:** 10.3390/cancers18071074

**Published:** 2026-03-26

**Authors:** Umar Rehman, Helen Hudson, Chung-Yu Hao, Yuju Ahn, Sanket Kachole, Jacklyn Liu, Shruti Patel, Mary Jue Xu, Maral J. Rouhani, Paul O’Flynn, Vaijayanti Pethe, Sheng-Po Hao, Dhananjay Kelkar, Matt Lechner

**Affiliations:** 1 Division of Surgery and Interventional Science, University College London, 43-45 Foley St, London W1W 7TY, UK; u.rehman@ucl.ac.uk (U.R.); s.kachole@ucl.ac.uk (S.K.);; 2 Department of Otolaryngology, Deenanath Mangeshkar Hospital & Research Centre, Erandwane, Pune 411004, India; 3 Department of Otolaryngology Head and Neck Surgery, Shin Kong Wu Ho-Su Memorial Hospital, No. 95, Wenchang Rd., Shilin District, Taipei City 111045, Taiwan; 4 School of Medicine, Fu-Jen University, Taiwan, 510 Zhongzheng Rd., Xinzhuang Dist., New Taipei City 242062, Taiwan; 5 Department of Audiology and Speech Language Pathology, Mackay Medical University, No. 46, Sec. 3, Zhongzheng Rd., Sanzhi Dist., New Taipei City 252005, Taiwan; 6 Cancer Institute, University College London, Paul O’Gorman Building, 72 Huntley St, London WC1E 6DD, UK; 7 Department of Otolaryngology-Head and Neck Surgery, University of California San Francisco, 2330 Post Street, San Francisco, CA 94115, USA; 8 Division of Medicine, University College London, 5 University Street, London WC1E 6JF, UK; 9 Head and Neck Centre and RNENT, University College London Hospitals NHS Foundation Trust, 47-49 Huntley St, London WC1E 6DG, UK

**Keywords:** betel nut, oral cancer, health education

## Abstract

**Introduction:** Globally, 600 million people chew areca nut, also known as betel nut, with habitual use being a major risk factor for oral cancer. Taiwan and India have some of the highest age-standardized incidence and mortality rates of oral cancer, underscoring the significant health burden in these countries. **Aims:** This observational cross-sectional study aims to investigate the betel nut consumption habits in the Taiwanese and Indian populations and assess their awareness of its association with oral cancer. **Methodology:** An interview was conducted and completed by 516 adults across Taiwan and 989 in India to assess betel nut habits and awareness of oral cancer risks. **Results:** In Taiwan, 95.2% (*n* = 491) were aware that betel nut can cause oral cancer versus 51.3% (*n* = 507) in India. Lifetime prevalence of betel nut use in Taiwan was 19.2% (*n* = 99) versus 42.6% (*n* = 421) in India. Only in India was betel nut chewing (OR: 0.6, 95% CI: 0.4–0.8, *p* < 0.001) associated with decreased awareness of the oral cancer risk linked to betel nut use. Using the population attributable fraction (PAF), an estimated 70.5% (95% CI: 46.0–85.5%) of oral cancer cases in India and 53.7% (95% CI: 42.2–64.1%) in Taiwan are potentially attributable to betel nut use. **Conclusions:** Betel nut use remains prevalent in India and Taiwan, with lack of awareness posing a greater challenge in India and betel nut use being associated with higher risks of self-reported oral cancer symptoms. Effective intervention requires country-specific strategies to curb usage, improve awareness of associated risks and government strategies to reduce betel nut cultivation and availability.

## Simple Summary

Betel nut, also called areca nut, is chewed by around 600 million people worldwide and is a major cause of oral cancer. This study looked at adults in Taiwan and India to understand their chewing habits and awareness of cancer risks. In Taiwan, most people (95%) knew that betel nut can cause oral cancer, while only half of Indian participants were aware. Chewing was more common in India (43%) than Taiwan (19%), and in India, chewing was linked to lower awareness of the cancer risk. Around half to two-thirds of oral cancer cases in these countries may be due to betel nut.

## 1. Introduction

Globally, up to 600 million people chew the areca nut [1]. The areca nut, commonly known as betel nut, is derived from the *Areca catechu* palm and is found in Asia, East Africa, and the Pacific Basin [1,2]. The areca nut is the primary ingredient in betel quid. It is typically consumed alongside betel leaves and a combination of spices, lime, or mixed with tobacco [2]. Betel nut is recognized as the fourth most used psychoactive substance worldwide [3], with studies showing that its use can lead to dependence [1,2,4,5].

Initially, there was insufficient evidence linking pure betel nut chewing with cancer, leading to the assumption that the carcinogenic effects were due to the tobacco added to the betel nut [2,6,7,8]. However, studies from Taiwan have shown that betel nut use alone, without tobacco, is linked to oral cancer [2]. The primary alkaloid in betel nut, arecoline, is known to induce carcinogenesis by causing DNA damage to epithelial cells and downregulating p53 [2]. Oral cancer is attributed to various risk factors; however, habitual betel nut chewing is strongly associated with the disease [2].

Oral cancer has been a significant public health concern in Asia, with the region experiencing the highest incidence, mortality, and national burden of the disease. For example, there has been a 177.5% increase in oral cancer cases in Asia between 1990 and 2019 [9,10,11,12,13]. Taiwan stands out globally as the only nation offering extensive, organized oral cancer screening programs on a nationwide scale [6,9]. This screening specifically targets individuals at high risk, such as betel quid chewers and smokers, and has been associated with a reduction in mortality rates, as well as identifying oral cancers at an earlier stage [6,9].

Taiwan and India rank amongst the top three nations with the highest age-standardized incidence and death rates of oral cancer [13], highlighting the significant burden and mortality of the disease in these countries and hence the focus of this study. Previous studies have highlighted a lack of awareness of the oral cancer risks associated with betel nut use among rural populations in India [4,14], but similar research has not been conducted in urban populations in India or the general population in Taiwan.

Due to the high incidence and mortality rates associated with oral cancer in Taiwan and India [13], this study aims to investigate the betel nut consumption habits in these populations and assess their awareness of its association with oral cancer.

## 2. Materials and Methods

### 2.1. In-Person Questionnaires

This was an observational cross-sectional study. In this study, 516 participants from the general population in Taipei, Taiwan, aged 18 and above, were invited to participate in an in-person interview that was conducted. Efforts were made to ensure the sample was age- and gender-balanced. Similarly, the survey was administered to 989 individuals in Pune and Delhi, India. In both locations, members of the public were approached and invited to participate by the study team and volunteers in-person in highly frequented public spaces (e.g., malls and supermarkets); individuals who agreed provided verbal consent before completing the questionnaire. Data collection was conducted over a two-week period as part of a public awareness campaign. The survey consisted of items relating to demographics, betel nut awareness, betel nut habits and self-reported symptoms of oral cancer. No personal or identifiable information was collected.

### 2.2. Eligibility

Participants were eligible to take part in the study if they were aged 18 years or older, able to provide informed consent, and agreed for their responses to be used for research purposes. Additionally, participants needed to be able to read and write in the local language: Hindi for participants in India and Mandarin Chinese for participants in Taiwan.

### 2.3. Statistical Analysis

Statistical analyses were conducted using *R* (version 4.5.3; R Foundation for Statistical Computing, Vienna, Austria) and *IBM SPSS Statistics* (version 31.0.2.0; IBM Corp., Armonk, NY, USA). Descriptive statistics were used to summarize participant demographics, awareness, and behaviors related to betel nut use.

Multivariable logistic regression models were fitted to identify factors associated with awareness of oral cancer risk and the presence of at least one oral cancer symptom, with results reported as odds ratios (ORs) and 95% confidence intervals (CIs). Models were implemented using the generalized linear model (GLM) framework with a binomial (logit) link function, adjusting for demographic variables and betel nut use (current and past).

Categories within age, income, and education variables were combined to ensure adequate sample sizes within groups and improve the stability of estimates. Prevalence extrapolations were calculated using recent national population estimates [15].

The Population Attributable Fraction (PAF) was calculated using Levin’s formula, as previously described [16]. Relative risks or odds ratios for oral cancer associated with areca nut use were obtained from published studies for India [17] and Taiwan [18]. The resulting PAFs were compared with previously reported estimates [16]. This approach assumes that published relative risk estimates are applicable to the populations studied.

### 2.4. Patient and Public Involvement

Patients and members of the public were not involved in the design of this research. This study was conducted as an anonymous, interviewer-administered cross-sectional survey in public settings as part of an awareness campaign.

## 3. Results

The study included *n* = 1505 adults, comprising *n* = 516 respondents from Taipei, Taiwan, and *n* = 989 respondents from Pune and Delhi, India. Detailed demographic characteristics for each sample are presented in Table 1 and Table 2.

### 3.1. Betel Nut Awareness

In Taiwan 95.2% (*n* = 491) were aware that betel nut use is associated with oral cancer versus 4.8% (*n* = 25) that did not know. In contrast, in India 48.7% (*n* = 482) were not aware that betel nut can cause oral cancer versus 51.3% (*n* = 507) that were aware of the association with oral cancer (Supplemental Figures S1 and S2).

### 3.2. Betel Nut Use in Taiwan and India

Lifetime prevalence of betel nut use in Taiwan was 19.2% (*n* = 99) versus 80.8% (*n* = 417) that had never used betel nut. Of those who have used betel nut, 28.3% (*n* = 28) were current users. Among those who had chewed betel nut, 30.3% (*n* = 30) started as a child or teenager.

Lifetime prevalence of betel nut use in India was 42.6% (*n* = 421) versus 57.4% (*n* = 568) that had never used betel nut. Of those that have chewed betel nut, 73.9% (*n* = 311) were current users. Among those who chewed betel nut 51.8% (*n* = 218) started as a child or teenager.

Using the population attributable fraction (PAF), an estimated 70.5% (95% CI: 46.0–85.5%) of oral cancer cases in India and 53.7% (95% CI: 42.2–64.1%) in Taiwan were attributable to betel nut use.

### 3.3. Symptoms of Oral Cancer

A total of 7.0% (*n* = 36) of respondents in Taiwan reported experiencing one or more symptoms of oral cancer, such as oral pain, white patches in the mouth, a lump in the throat or mouth, non-healing oral ulcers, tongue pain during movement, or jaw pain during movement. Among those with symptoms, 47.4% reported having them for more than two weeks. In comparison, 8.9% (*n* = 88) of respondents in India reported having one or more symptoms of oral cancer, with 44.7% experiencing symptoms for more than two weeks (Supplemental Figures S3 and S4).

### 3.4. Logistic Regression

Logistic regression analysis was performed with betel nut awareness and with having one or more symptoms of oral cancer as outcome variables. This analysis was conducted separately for the Indian and Taiwanese populations. In Taiwan, no statistically significant associations were observed between betel nut consumption and awareness of oral cancer risk (Table 3), whilst for the Indian population, betel nut chewing (OR: 0.6, 95% CI: 0.4–0.8, *p* < 0.001) and tobacco chewing (OR: 0.5, 95% CI: 0.3–0.7, *p* < 0.001), were associated with decreased awareness of the risk of oral cancer with betel nut use (Table 4).

An increased likelihood of having one or more symptoms of oral cancer in Taiwan was observed among individuals who chewed betel nut (OR: 11.5, 95% CI: 3.3–45.3, *p* < 0.001), were male (OR: 10.3, 95% CI: 2.7–59.9, *p* = 0.002), or chewed tobacco (OR: 13.6, 95% CI: 2.3–88.0, *p* = 0.004) (Supplemental Table S1). Similarly, in the Indian population, oral cancer symptoms were associated with betel nut chewing (OR: 2.9, 95% CI: 1.6–5.1, *p* < 0.001), tobacco chewing (OR: 6.2, 95% CI: 3.4–11.8, *p* < 0.001), male gender (OR: 2.0, 95% CI: 1.0–4.3, *p* = 0.047) and tobacco smoking (OR: 3.2, 95% CI: 1.9–5.5, *p* < 0.001) (Supplemental Table S2).

### 3.5. Comparison of Betel Nut Usage Between 1992 and 2021 in Taiwan

This study compared the lifetime prevalence of betel nut use between the rates reported by Ko et al. (1992) over 30 years ago [19]. A chi-squared test revealed a significant increase in prevalence (χ^2^ = 28.8, *p* < 0.0001). The odds of lifetime betel nut use were more than doubled in the last 30 years in Taiwan (OR: 2.2, 95% CI: 1.6–2.9, *p* < 0.001).

## 4. Discussion

### 4.1. Key Results

In this study, it was found that 19.2% of people in Taiwan and 42.6% in India have used or are currently using betel nut which, when extrapolated to national populations, corresponds to approximately 4.5 million lifetime users in Taiwan and 620.4 million in India [15]. Awareness of the link between betel nut use and oral cancer varies significantly between the two nations, with 95.2% of individuals in Taiwan being aware of the risks compared to only 51.3% in India.

Individuals with a history of betel nut use, whether past or current, were more likely to report symptoms of oral cancer. Moreover, in India, those who had used betel nut, either currently or in the past, were less likely to be aware of its link to oral cancer, a trend that was not observed in Taiwan. Despite the higher awareness levels in Taiwan, the prevalence of betel nut usage has persisted and even increased significantly over the last 30 years [19].

### 4.2. Comparison with Previous Literature

The substantial disparity in awareness between the nations may be attributed to Taiwan’s national oral cancer screening program, which has been associated with earlier detection and treatment of oral cancer [9]. By contrast, India lacks similar nationwide initiatives, consistent with previous research showing substantial knowledge gaps among Indian populations both locally but also amongst migrant Indian communities [4,14,20,21,22].

Although prior studies have highlighted the addictive properties of betel nut, as well as societal misconceptions and perceived medicinal benefits, as barriers to cessation, this study does not directly assess addiction, dependence, or cessation behaviors [1,4,20]. Nonetheless, the observed differences in awareness suggest that cultural and structural factors may influence usage patterns and the potential effectiveness of public health campaigns, even where knowledge of cancer risk is high.

### 4.3. Interpretation

The challenges and drivers associated with betel nut use likely differ between Taiwan and India. Understanding these differences will help inform nation-specific strategies to reduce usage. In Taiwan, where awareness is high, interventions should target dependence, cultural practices and behavioral counseling. Efforts to reduce cultivation and provide cessation support have faced systemic challenges, including resistance from farmers and limited uptake of cessation programs.

In contrast, India faces a different set of challenges and the priority should involve closing the knowledge gap through educational campaigns targeting both adults and young people. As early exposure was common, with many betel nut users reporting to have commenced betel nut early in childhood or adolescence, this underscores the need for school and community-based interventions [4,21,22,23,24,25,26]. Awareness programs could provide a solid foundation for future initiatives such as oral cancer screening, which is shown to be cost-effective in high-risk populations [22].

Moreover, early initiation of betel nut use is not unique to Taiwan and India, but has been widely reported across Asia. Studies from Pakistan, for example, indicate that 30–74% of children in Karachi have used betel nut, with early consumption strongly linked to limited regulation, social norms and family influence [27]. Parental and household use were identified as key determinants of children’s initiation [27]. These findings suggest that effective prevention strategies must extend beyond individual-level awareness and include parents, teachers and local communities. Such multi-level interventions may help discourage early uptake not only in Taiwan and India but also in other regions with high usage across South Asia and beyond. Together, these patterns of early use highlight the need to consider the structural availability of betel nut, which continues to shape consumption behaviors across both countries.

The easy availability and supply of betel nut further complicate control efforts. In Taiwan, betel nut remains a major agricultural product [17]. Although government measures such as incentivizing farmers to grow alternative crops and converting betel nut plantations aim to curb cultivation, progress has been slow. Resistance from farmers, delays in implementing regulations, and policymakers’ reluctance to propose drastic changes due to the crop’s economic importance have hindered efforts [17]. The persistence of betel nut usage reflects not only cultural attachment but also systemic challenges in reducing its availability.

Meanwhile in India, betel nut cultivation has expanded significantly, rising from 250,000 tons annually in the early 1900s to 900,000 tons in 2020 [3]. This growth has been fueled by the absence of national legislation to regulate production and by the lucrative profits it generates for farmers. Prices increased from $2.7–$3.4 per kilogram in 2019 to $6.7–$7.1 per kilogram in 2021, incentivizing further cultivation [20]. Additionally, Indian policies, such as tax incentives, have supported its trade, further boosting its domestic supply and catering for the demand [20]. From the current study, it was not possible to assess whether ease of access contributes to the high usage rates observed. However, prior research suggests that structural factors, such as availability and cultivation, may play an important role in shaping consumption patterns. In this context, this study suggests that policies focused on awareness and behavioral interventions may have the potential to reduce betel nut use, while strategies aimed at limiting availability and cultivation could provide additional benefits. Nevertheless, further research is needed to evaluate the effectiveness of these approaches [4].

Taiwan has made efforts to reduce production and raise awareness of the risks of betel nut, yet its usage persists. Hence, dependence may play a part in usage habits. Betel nut dependence, often overlooked, is a significant challenge in both Taiwan and India, with studies indicating it can exceed dependence on alcohol [1,21]. Withdrawal symptoms often act as a barrier to cessation, driven by the effects of arecoline, the primary psychoactive component of betel nut [1,22]. Arecoline’s monoamine oxidase-A inhibitor-like properties increase dopamine and serotonin levels, reinforcing dependence. However, many cessation programs overlook the biological and psychological aspects of addiction, as well as the importance of individual behavioral counseling [22]. Unlike smoking or alcohol cessation, where pharmaceutical agents are often available, there is a lack of targeted treatments for betel nut dependence. Early research suggests that certain medications, such as MAO-A inhibitors or non-selective serotonin reuptake inhibitors, could have potential, but further clinical trials are needed to validate their efficacy [22,23]. Moreover, alongside oral cancer awareness and association with betel nut use, efforts should be made to highlight long-term dependency associated with betel nut usage.

### 4.4. Generalizability

This study highlights the need for tailored strategies in each country. While Taiwan’s efforts should focus on addressing dependence and cultural influences, India must first close the knowledge gap through awareness campaigns [4,14,18,19,20,21,22,23,24,25,26]. This will create a foundation for further interventions in India, including oral cancer screening, which has been shown to be cost-effective in high-risk groups [19], as well as managing betel nut dependence [4]. Moreover, a large proportion of betel nut users, both past and present, in both nations reported using betel nut as children or teens. Further work is needed to address this issue, likely through collaboration with schools and local communities to reach younger users to provide education and prevent usage from an early age.

Although this study focused on Taiwan and India, the high prevalence observed in India mirrors betel nut use patterns across neighboring South Asian countries. Bangladesh reports population use estimates of 29.3–33.6% and Sri Lanka approximately 23.4%, indicating that the issue extends beyond national boundaries [28,29]. These similarities suggest that interventions developed for India, such as community-based awareness efforts, school prevention programs and regulation of betel nut products, may also be applicable across the wider South Asian region. This regional perspective highlights the need for cross-country collaboration and harmonized public health strategies to address the shared burden of betel nut-related oral cancer.

### 4.5. Limitations

Several limitations of the study should be addressed. Firstly, the use of convenience sampling in public locations such as shopping centers and supermarkets introduces potential selection bias and limits the generalizability of findings. Although efforts were made to recruit a diverse sample, the study populations were restricted to urban centers— Pune and Delhi in India, and Taipei in Taiwan—and may not reflect patterns in rural areas or other regions with differing cultural practices and prevalences of betel nut use. As such, the findings should be interpreted as representative of urban populations rather than national estimates. Secondly, oral cancer-related symptoms were self-reported and not clinically verified. This introduces the possibility of recall bias and misclassification bias, as these symptoms are not specific to oral cancer and may be attributable to other benign conditions. Clinical examination was not feasible due to the study’s design in public, non-clinical settings, where ensuring participant privacy and appropriate clinical standards would be challenging. Additionally, the survey did not include questions regarding current or past diagnoses or treatment of oral cancer. Furthermore, the PAF calculations were based on relative risk estimates obtained from external studies rather than derived from the current dataset. This assumes that these estimates are generalizable to the study populations, which may not fully account for regional differences in exposure patterns, co-risk factors, and healthcare access. Therefore, the PAF estimates should be interpreted as approximate indicators of population-level burden rather than precise measures. A further potential limitation is the imbalance in age distribution between the study populations, with a higher proportion of younger adults in India compared with Taiwan, which may have influenced the findings as younger individuals may differ in health awareness and engagement with health information.

## 5. Conclusions

Betel nut use remains high in both nations, associated with the burden of oral cancer. Despite awareness of its risks amongst the Taiwanese population, many continue using it, possibly due to dependence and cultural significance. In India, usage is likely also driven by a lack of awareness of cancer risks. Both nations struggle with its widespread availability, exacerbating usage, and the significant economic burden of betel-nut induced oral cancer. This study highlights the need for context-specific approaches that may help reduce betel nut consumption, including improving awareness, addressing dependence, and considering broader structural factors such as availability. Further research is required to evaluate the effectiveness of such strategies.

## Figures and Tables

**Table 1:** Demographic characteristics of the Taiwan sample.

Taiwan Population	Total (*N* = 516)
**Age**	
18–29	59 (11.4%)
30–39	65 (12.6%)
40–49	128 (24.8%)
50–59	159 (30.8%)
60–69	92 (17.8%)
>70	13 (2.5%)
**Gender**	
Female	261 (50.6%)
Male	255 (49.4%)
**Marital status**	
Single	146 (28.3%)
Married/civil partnership	339 (65.7%)
Divorced/separated	31 (6.0%)
**Education**	
None	3 (0.6%)
Primary school	12 (2.3%)
Upper primary school	14 (2.7%)
Secondary school	69 (13.4%)
Higher secondary school	76 (14.7%)
College or higher	342 (66.3%)
**Income (NT$)**	
Less than 20,000	195 (37.8%)
20,000–34,999	143 (27.7%)
35,000–49,999	63 (12.2%)
50,000–74,999	39 (7.6%)
75,000–99,999	26 (5.0%)
More than 100,000	50 (9.7%)
**Current or previous use of betel nut**	
No	417 (80.8%)
Yes	99 (19.2%)
**Tobacco chewing**	
No	504 (97.7%)
Yes	12 (2.3%)

**Table 2:** Demographic characteristics of India sample.

India Population	Total (*N* = 989)
**Age**	
18–29	483 (48.8%)
30–39	218 (22.0%)
40–49	138 (14.0%)
50–59	58 (5.9%)
60–69	55 (5.6%)
>70	37 (3.7%)
**Gender**	
Female	321 (32.5%)
Male	668 (67.5%)
**Marital status**	
Not married	404 (40.8%)
Married	585 (50.2%)
**Education**	
None	12 (1.2%)
Primary school	79 (8.0%)
Upper primary school	113 (11.4%)
Secondary school	88 (8.9%)
Higher secondary school	171 (17.3%)
College or higher	526 (53.2%)
**Current or previous use of betel nut**	
No	568 (57.4%)
Yes	421 (42.6%)
**Tobacco chewing**	
No	823 (83.2%)
Yes	166 (16.8%)

**Table 3:** Logistic regression for betel nut awareness in Taiwan.

	OR	95% CI	*p*-Value
**Age**			
Young (18–39)	—	—	—
Middle-age (40–59)	1.1	0.3, 3.4	0.9
Old (>60)	1.3	0.3, 6.3	0.7
**Gender**			
Female	—	—	—
Male	1.5	0.6, 3.8	0.4
**Marital status**			
Single	—	—	—
Married/civil partnership	2.0	0.7, 5.7	0.2
Divorced/separated	1.03	0.2, 8.0	>0.9
**Tobacco smoking**			
No	—	—	—
Yes	2.1	0.5, 10.8	0.3
**Tobacco chewing**			
No	—	—	—
Yes	0.3	0.1, 2.8	0.2
**Education**			
Below	—	—	—
College or higher	2.6	1.0, 7.2	0.06
**Income**			
Low (<35,000)	—	—	—
Middle (35,000–74,999)	0.5	0.2, 1.5	0.2
High (>74,999)	0.5	0.1, 2.5	0.3
**Betel nut chewing**			
No	—	—	—
Yes	0.4	0.1, 2.2	0.2

OR = Odds Ratio, CI = Confidence Interval.

**Table 4:** Logistic regression of betel nut awareness in India.

	OR	95% CI	*p*-Value
**Age**			
Young (18–39)	—	—	—
Middle-age (40–59)	0.6	0.4, 0.8	0.004
Old (>60)	0.34	0.2, 0.6	<0.001
**Gender**			
Female	—	—	
Male	1.3	1.0, 1.8	0.05
**Marital status**			
Not married	—	—	
Married	1.2	0.9, 1.6	0.3
**Tobacco smoking**			
No	—	—	—
Yes	1.1	0.8, 1.6	0.6
**Tobacco chewing**			
No	—	—	—
Yes	0.5	0.3, 0.7	<0.001
**Education**			
Below	—	—	—
College or higher	1.3	1.0, 1.7	0.08
**Betel nut chewing**			
No	—	—	—
Yes	0.6	0.4, 0.8	<0.001

OR = Odds Ratio, CI = Confidence Interval.

## Data Availability

Data is available upon reasonable request to the corresponding author within the limits of the ethical approval.

## Supplementary Materials

The following supporting information can be downloaded at: https://www.mdpi.com/article/10.3390/cancers18071074/s1, Figure S1: Awareness of oral cancer risk with betel nut usage in Taiwan stratified by betel nut and tobacco use; Figure S2: Awareness of oral cancer risk with betel nut usage in India stratified by betel nut and tobacco use; Figure S3: Oral cancer symptoms in Taiwan stratified by betel nut and tobacco use; Figure S4: Oral cancer symptoms in India stratified by betel nut and tobacco use; Table S1: Logistic regression for one or more symptoms of oral cancer in Taiwan; Table S2: Logistic regression for one or more symptoms of oral cancer in India.
