# Supplementary material for: Lifetime Prevalence of Betel Nut Chewing in India and Taiwan: Raising Awareness of Oral Cancer Risks and the Urgent Call for Regulation
Source: Cancers (Basel). 2026 Mar 26;18(7):1074. doi: 10.3390/cancers18071074 (PMC13072213; doi:10.3390/cancers18071074)
Supplement: Supplementary file 1 [file cancers-18-01074-s001.zip › cancers-4195735-supplementary.pdf]

Supplemental Material

Supplemental Table S1. Logistic regression for one or more symptoms of oral cancer in Taiwan.

	**OR**	**95% CI**	**p-value**
**Age**			
Young (18-39)	—	—	
Middle-age (40-59)	1.7	0.4, 8.6	0.5
Old (>60)	0.8	0.1, 5.8	0·8
**Gender**			
Female	—	—	
Male	10.3	2.7, 59.9	0.002
**Marital status**			
Single	—	—	
Married/civil partnership	0.62	0.2, 2.2	0.4
Divorced/separated	2.6	0.5, 13.4	0.2
**Tobacco smoking**			
No	—	—	
Yes	2.4	0.8, 6.9	0.09
**Tobacco chewing**			
No	—	—	
Yes	13.6	2.3, 88.0	0.004
**Education**			
Below	—	—	
College or higher	0.1	0.0, 0.3	< 0.001
**Income**			
Low (<35,000)	—	—	
Middle (35,000-74,999)	0.7	0.1, 3.7	0.7
High (>74,999)	2.9	0.5, 15.2	0.2
**Betel nut chewing**			
No	—	—	
Yes	11.5	3.3, 45.3	< 0.001
OR = Odds Ratio, CI = Confidence Interval			

Supplemental Table S2. Logistic regression for one or more symptoms of oral cancer in India

	OR	95% CI	p-value
**Age**			
Young (18-39)	—	—	—
Middle-age (40-59)	2.7	1.5, 5.0	0.002
Old (>60)	0.9	0.2, 2.6	0.8
**Gender**			
Female	—	—	—
Male	2.0	1.0, 4.3	0.05
**Marital status**			
Not married	—	—	—
Married	0.6	0.3, 1.1	0.08
**Tobacco smoking**			
No	—	—	—
Yes	3.2	1.9, 5.5	< 0.001
**Tobacco chewing**			
No	—	—	—
Yes	6.2	3.4, 11.8	< 0.001
**Education**			
Below	—	—	—
College or higher	0.8	0.5, 1.5	0.5
**Betel nut chewing**			
No	—	—	—
Yes	2.9	1.6, 5.1	< 0.001
OR = Odds Ratio, CI = Confidence Interval			

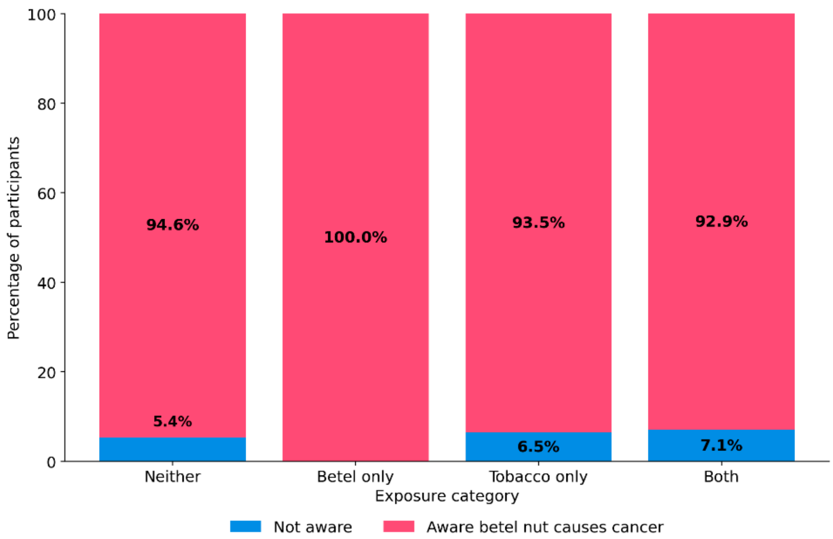

Supplemental Figure S1. Awareness of oral cancer risk with betel nut usage in Taiwan stratified by betel nut and tobacco use.

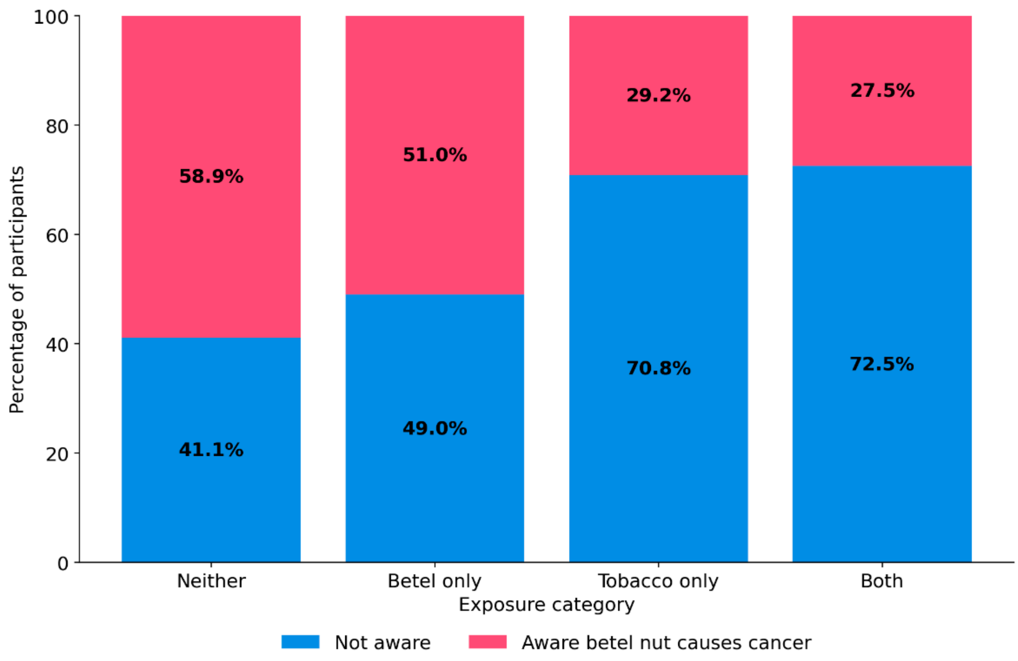

Supplemental Figure S2. Awareness of oral cancer risk with betel nut usage in India stratified by betel nut and tobacco use.

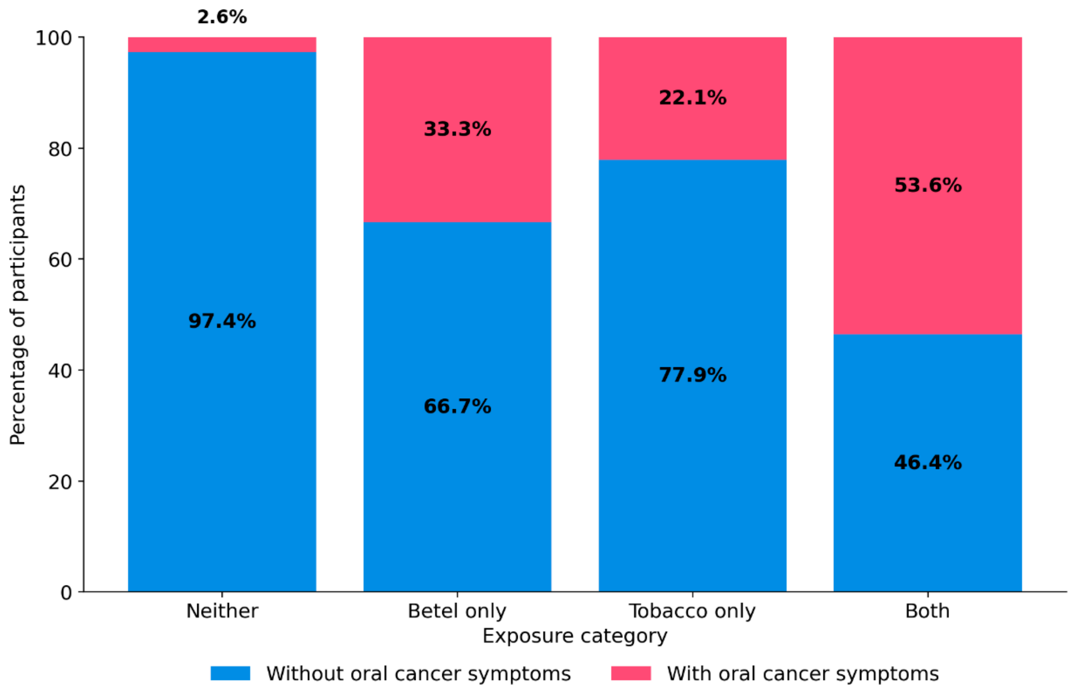

Supplemental Figure S3. Oral cancer symptoms in Taiwan stratified by betel nut and tobacco use.

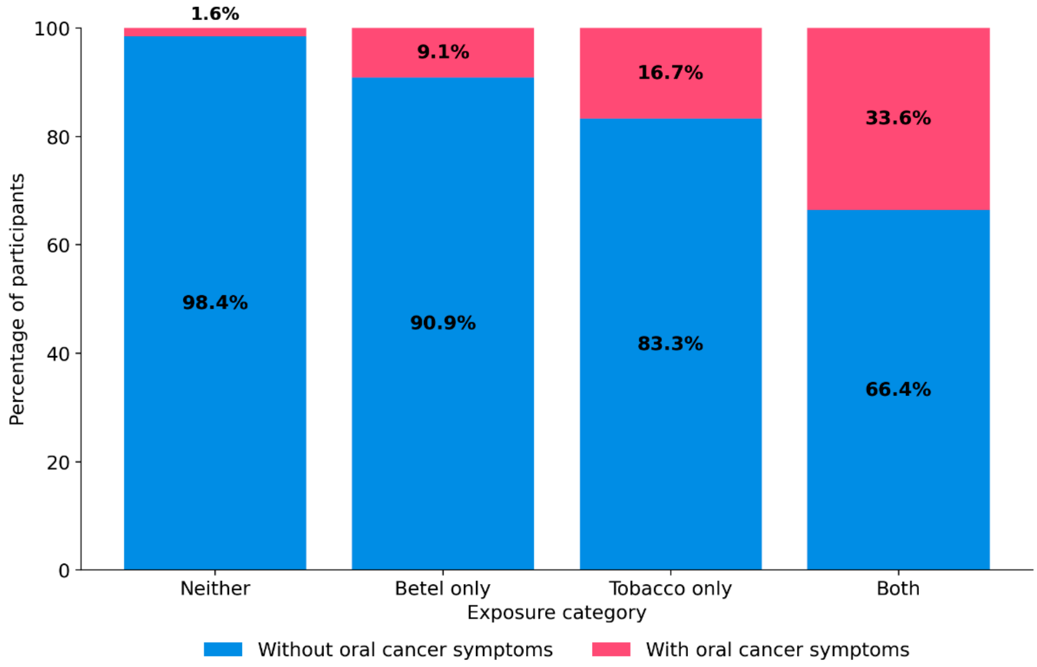

Supplemental Figure S4. Oral cancer symptoms in India stratified by betel nut and tobacco use.
